# Utilisation of and factors associated with non-COVID-19 healthcare services in public facilities amongst cross-border migrants in Thailand, 2019–2022

**DOI:** 10.1186/s12889-024-17657-0

**Published:** 2024-01-09

**Authors:** Saruttaya Wongsuwanphon, Krittinan Boonrumpai, Chinnakrit Sangvisut, Yanisa Hattasarn, Suphanat Wongsanuphat, Rapeepong Suphanchaimat

**Affiliations:** 1 Department of Social Medicine, Pranangklao Hospital, Ministry of Public Health, Nonthaburi, Thailand; 2 Division of Epidemiology, Department of Disease Control, Ministry of Public Health, Nonthaburi, Thailand; 3 Department of Computer Engineering, Faculty of Engineering, King Mongkut’s University of Technology Thonburi, Bangkok, Thailand; 4 Princess Srisavangavadhana College of Medicine, Chulabhorn Royal Academy, Bangkok, Thailand; 5 International Health Policy Program (IHPP), Ministry of Public Health, Nonthaburi, Thailand

**Keywords:** Migrants, COVID-19, Negative binomial regression, Health service, Thailand

## Abstract

**Background:**

It is believed that the COVID-19 pandemic might disrupt routine healthcare services. A vulnerable group such as cross-border migrants is of critical concern if the pandemic affects their service utilisation. In this study, we aim to explore the impact of COVID-19 and other related factors on non-COVID-19 service amongst cross-border migrants in Thailand.

**Methods:**

We conducted an ecological time-series cross-sectional analysis using secondary data from 2019 to 2022, focusing on insured and non-insured migrants in a unit of a provincial monthly quarter. We obtained data on registered migrants from the Ministry of Labour and inpatient visits from the Ministry of Public Health (MOPH). Our analysis involved descriptive statistics and a random-effects negative binomial regression, considering variables such as COVID-19 cases, number of hospital beds, registered regions, and COVID-19 waves. We assessed inpatient utilisation number and rate as our primary outcomes.

**Results:**

The admission numbers for insured and non-insured migrants in all regions increased 1.3–2.1 times after 2019 despite a decrease in the numbers of registered migrants. The utilisation of services for selected communicable and non-communicable diseases and obstetric conditions remained consistent throughout 2019–2022. The admission numbers and rates were not associated with an increase in COVID-19 incidence cases but significantly enlarged as time passed by compared to the pre-COVID-19 period (44.5–77.0% for insured migrants and 15.0–26.4% for non-insured migrants). Greater Bangkok saw the lowest admission rate amongst insured migrants, reflected by the incidence rate ratio of 5.7–27.5 relative to other regions.

**Conclusion:**

The admission numbers and rates for non-COVID-19 healthcare services remained stable regardless of COVID-19 incidence. The later pandemic waves (Delta and Omicron variants) were related to an increase in admission numbers and rates, possibly due to disruptions in outpatient care, leading to more severe cases seeking hospitalisation. Lower admission rates in Greater Bangkok may be linked to the fragmentation of the primary care network in major cities and the disintegration of service utilisation data between private facilities and the MOPH. Future research should explore migrant healthcare-seeking behaviour at an individual level, using both quantitative and qualitative methods for deeper insights.

**Supplementary Information:**

The online version contains supplementary material available at 10.1186/s12889-024-17657-0.

## Introduction

Thailand is one of the common destination countries for cross-border migrants in Southeast Asia. Its higher wage relative to its neighbouring countries, in combination with the domestic labour shortage, greatly attract international migrant workers. The majority of migrants are from Cambodia, Lao PDR, Myanmar, and Vietnam (CLMV). Due to the high demand for labour, the Thai Government always employs leniency policies by allowing undocumented migrants to work in the country legitimately, conditional upon registration with the Government [1]. The International Organisation for Migration recently reported that the prevalence number of migrant workers in Thailand (encompassing those legally imported from neighbouring countries and the ex-illegal migrants who later registered with the government) was approximately 3 million [2]. This figure has already excluded undocumented migrants, whose exact figure is unknown. The latest meta-analysis study by the Thai Ministry of Public Health (MOPH) suggests that about 29.6% of all migrants are undocumented, with a 95% confidence interval (95% CI) ranging from 20.1 to 40.0% [3].

Upon registration, an undocumented migrant will become documented and acquire a work permit (issued by the Ministry of Labour [MOL]) and have their profile included in the civil registry (by the Ministry of Interior). Alongside the issuance of civil registry and work permit, registered migrants will be insured by either one of the two main public insurance schemes: the Social Security Scheme (SSS) for formal workers (managed by the MOL) or the Health Insurance Card Scheme for informal workers (managed by the MOPH). Details of both schemes are demonstrated in Supplementary File 1 [4, 5].

Thailand is amongst several countries that have been severely affected by the Coronavirus Disease 2019 (COVID-19). The first wave of the pandemic started in early 2020, followed by the second wave emerging in late 2020 and lasting till early 2021. The epi-centre of the second wave was around the inner-city in a vicinity province of Bangkok where migrants are highly populated. The third wave (the Alpha variant) began in April 2021 and was soon followed by the fourth wave in June 2021 due to a massive widespread of the Delta variant. The last wave, caused by the Omicron variant, occurred since the inception of 2022. Then, the Thai Government declared the end of the outbreak and considered COVID-19 as an endemic disease [6].

During the pandemic, there was a remarkable concern if and to what extent the routine services would be disrupted, resulting in an unmet need for healthcare services in the Thai population. Pitayarangsarit et al. conducted a web-based survey on 169 health facilities’ directors in Bangkok. They found that Bangkok’s healthcare services for non-communicable diseases were critically disrupted during the pandemic [7]. A multi-country study by Reddy pointed to a slight increase (2.73%) in the monthly volume of newborn and maternity care in Thailand during 2020 compared to the average monthly volume a year before [8]. Huabbangyang et al. reported a 9.36% rise in the number of emergency medical service patients in Bangkok in 2020, relative to the corresponding number in 2019 [9].

Despite the existence of some studies mentioned above, those pieces of evidence analysed the influence of COVID-19 on the healthcare service volume in the entire population, while research focusing on this issue amongst migrant workers is still lacking. As cross-border migrants are amongst the most vulnerable and neglected members of society (even in the non-pandemic situation), we thus aim to explore the impact of COVID-19 and other related factors on non-COVID-19 service volume and utilisation amongst cross-border migrants in Thailand.

## Methods

### Study design and data sources

**Fig. 1:**
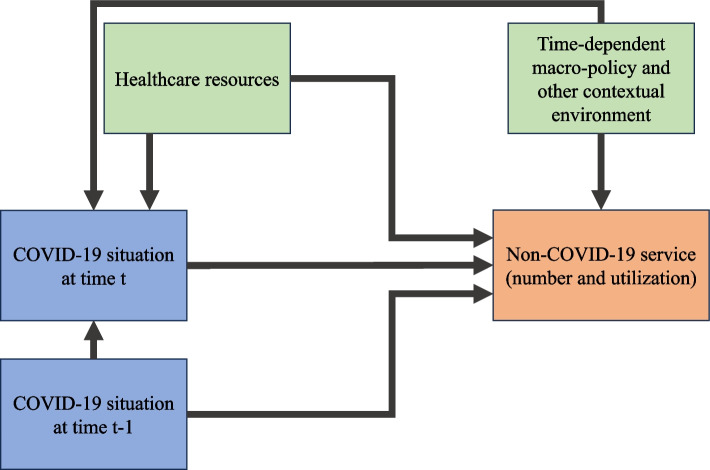
Analysis framework

We used secondary data analysis based on the data routinely collected in the Thai healthcare system. An ecological time-series cross-sectional data analysis was employed. In this study, we focused only on inpatient (IP) care as we presumed that IP utilisation better reflects healthcare need than outpatient (OP) care, especially in critical illnesses. For population scope, we confined the analysis to CLMV migrant workers utilising MOPH health facilities. As registered migrant workers must be above 15 years of age, migrants aged below 15 years were excluded. We hypothesised that the COVID-19 service burden (as reflected by the incidence number of COVID-19 cases in the country, both Thai and non-Thai patients) might cause constraints to the healthcare system, ultimately entailing the change in non-COVID-19 service volume and utilisation amongst migrants. Also, the influence of the COVID-19 service burden might have a lag-time effect. That is, the burden in the past might have some impact on the healthcare system at the present. We defined a non-COVID-19 admission as an admission where its principal diagnosis was not coded with any of the following International Classification of Diseases 10th Revision (ICD-10) codes: U07.1 (COVID-19, virus identified), U07.2 (COVID-19, virus not identified), and U29.0 (isolation). The ICD-10 codes directly related to non-illness healthcare seeking, such as Z00.0 (general medical check-up) and Z02.7 (issuance of medical certificate), were also excluded. We later explored if there was any change in specific principal diagnoses by using the following diagnoses as proxies: (i) communicable diseases that need continuing treatment (ICD-10 coded as A15-A19 [tuberculosis], B18 [chronic viral hepatitis], B20-B24 [HIV disease], and A50-A64 [infections with a predominantly sexual mode of transmission], (ii) key non-communicable diseases (ICD-10 coded as I10-I15 [hypertensive diseases], I20-I25 [ischaemic heart diseases], I48 [atrial fibrillation and flutter], I50 [heart failure], I64 [stroke], I69.3-I69.4 [sequelae of cerebral infarction and sequelae of stroke] and N18.4-N18.5 [stage-4 and stage-5 chronic kidney diseases]), and (iii) the obstetric conditions (ICD-10 codes beginning with letter ‘O’). Other factors, such as pandemic waves and regions of residence, were included in the analysis to address time-dependent macro-policy and spatial contextual environment. We classified the pandemic into five waves based on the predominant SARS-CoV-2 antigenic strains in the population: (i) pre-COVID-19 (throughout 2019), (ii) wild type (throughout 2020), (iii) Alpha variant (first and second quarters of 2021), (iv) Delta variant (third and fourth quarters of 2021), and (v) Omicron variant (throughout 2022). The data were arranged in a province-quarter format. The analysis idea was based on the following framework, Fig. 1. Table 1 displays the list of variables used in the analysis and a key description of the variables and data sources.

**Table 1:** Key variables used in the analysis

Variables	Rationale for inclusion	Data source	Remark	Unit
Incidence number of non-COVID-19 admissions (made by both insured and non-insured migrants)	As dependent variable	Health Data Centre, Office of Permanent Secretary, Ministry of Public Health	Equalling all admissions where the principal diagnosis was not coded with U07.1, U07,2, and Z290 according to the International Classification of Diseases 10th Revision (ICD-10)	Admissions
Prevalence number of registered migrants	To calculate admission rate amongst insured migrants	Foreign Workers Administration Office, Department of Employment, Ministry of Labour	The quarterly prevalence number of registered migrants was acquired by taking a mean of the monthly prevalence number in three consecutive months	Persons
The admission rate of non-COVID-19 care amongst insured migrants	As dependent variable	Health Data Centre, Office of Permanent Secretary, Ministry of Public Health and Foreign Workers Administration Office, Department of Employment, Ministry of Labour	Number of admissions by insured migrants per number of total registered migrants (assume all registered migrants are insured)	Admissions per person
COVID-19 incidence number in quarter t	As independent variable	Division of Epidemiology, Department of Disease Control, Ministry of Public Health	Cumulative incidence number of COVID-19 cases in a particular quarter	1,000 persons
COVID-19 incidence number in quarter t-1	To capture the lag-time effect of the independent variable on the outcome	Division of Epidemiology, Department of Disease Control, Ministry of Public Health	Cumulative incidence number of COVID-19 cases in a quarter before	1,000 persons
Number of hospital beds in a province	As a proxy for healthcare resources	Health Administration Division, Office of Permanent Secretary, Ministry of Public Health [10], and Office of the National Economic and Social Development Council [11]	Assume a constant monthly bed number throughout a year	1,000 beds
Region (Greater Bangkok, North, Northeast, Central, and South)	As a proxy for idiosyncratic spatial healthcare management	-	Greater Bangkok comprising Bangkok, Nakhon Pathom, Nonthaburi, Pathum Thani, Samut Prakan, and Samut Sakhon	Dimensionless
Pandemic	As a proxy for time-dependent macro-policy and contextual environment in response to different phases of the outbreak	-	-	(i) pre-COVID-19 (2019), (ii) wild type (2020), (iii) Alpha variant (first and second quarters of 2021), (iv) Delta variant (third and fourth quarters of 2021), and (v) Omicron variant (2022)

### Statistical analysis

We started by describing the frequency of non-COVID-19 admissions (sorted by the insured status of the patients), COVID-19 incidence number, volume of registered migrants across space (regions) and times (quarter-years), admission rate for non-COVID-19 service amongst insured migrants (assume all migrants registered with the MOL were insured), and percentage distribution of communicable, non-communicable diseases, and obstetric conditions from all non-COVID-19 admissions by descriptive statistics. Later, a random-effects negative binomial regression was performed in three different models, varying by three different outcomes: (i) non-COVID-19 admission number amongst insured migrants, (ii) non-COVID-19 admission number amongst non-insured migrants, and (iii) non-COVID-19 admission rate amongst insured migrants. It is worth noting that since there was no official report on the exact number of existing undocumented (non-insured) migrants, the admission rate amongst non-insured migrants was omitted. From a methodologic point of view, we performed negative binomial regression instead of traditional Poisson regression to account for the over-dispersion nature of the outcome variables. Stata v15.1 (serial number 301506215585) was used for all statistical analyses. Incidence rate ratio (IRR) and 95% confidence interval (CI) were demonstrated. Rstudio v4.2.2 was used for data visualisation.

### Ethics consideration

This study was part of the monitoring of the healthcare system performance as per the mandate of the International Health Policy Program (IHPP), the MOPH. Further, we used only secondary data from public sources. In this regard, no ethics clearance was required. Nonetheless, we followed the ethical standards for research as stipulated by the Declaration of Helsinki. All individual information in the datasets was kept anonymous. All findings will be presented and utilised only for academic reasons.

## Results

### Descriptive statistics

**Fig. 2:**
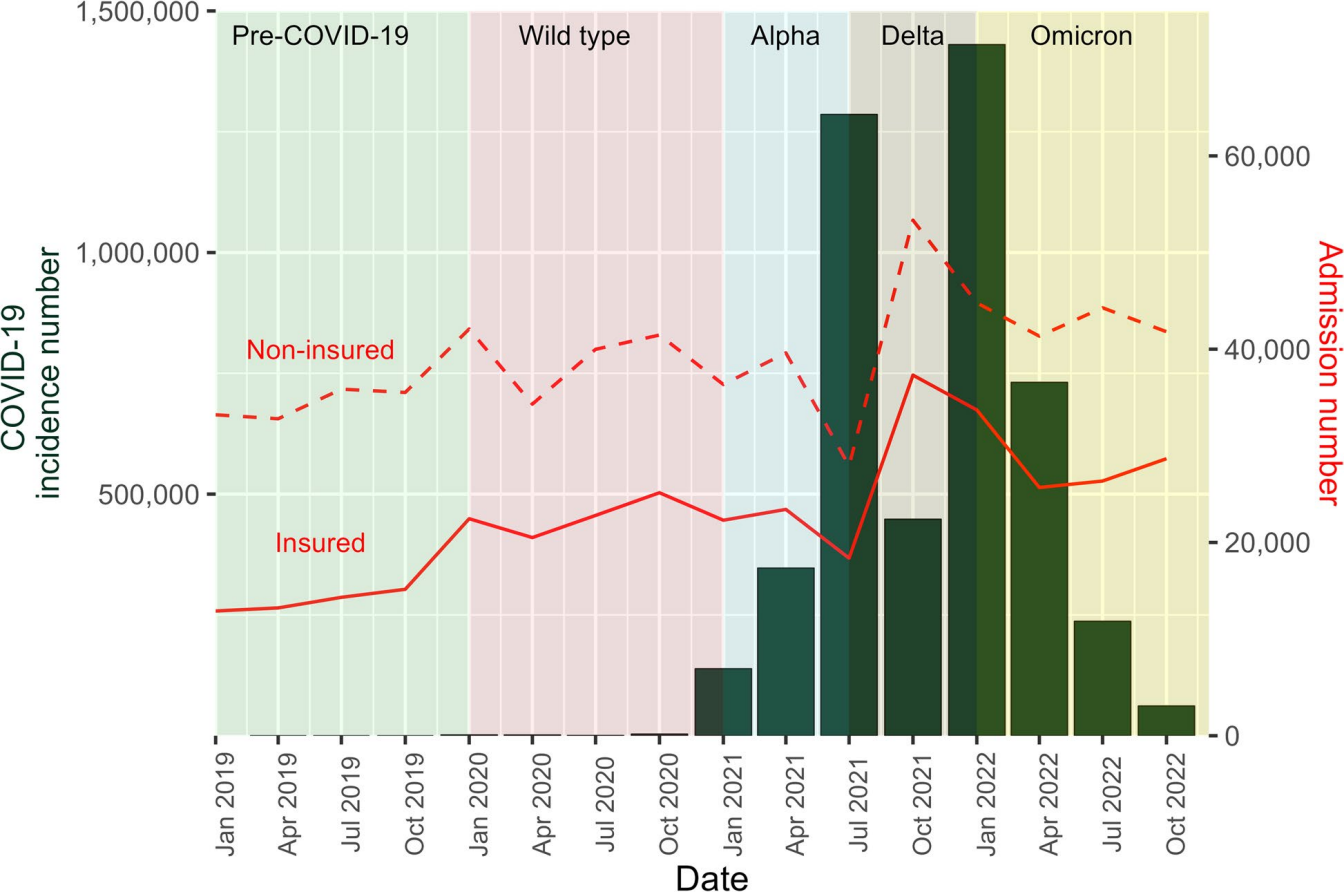
COVID-19 incidence and admission number for non-COVID-19 services by insured and non-insured migrants, 2019–2022

The COVID-19 outbreak in Thailand commenced in the first quarter of 2020, with incidence numbers ranging from 389 to 3,309 cases per quarter for that year. Subsequently, incidence numbers surged during the transition periods between each wave, peaking at 138,596, 1,285,854, and 1,430,280 during the early phases of the Alpha, Delta, and Omicron variants, respectively. These numbers then decreased to 61,488 in the final quarter of 2022. The admission numbers for both insured and non-insured migrants exhibited a similar temporal pattern. They gradually increased in late 2019, experienced a slight drop during the late Alpha wave, and peaked during the Delta wave, Fig. 2.

**Fig. 3:**
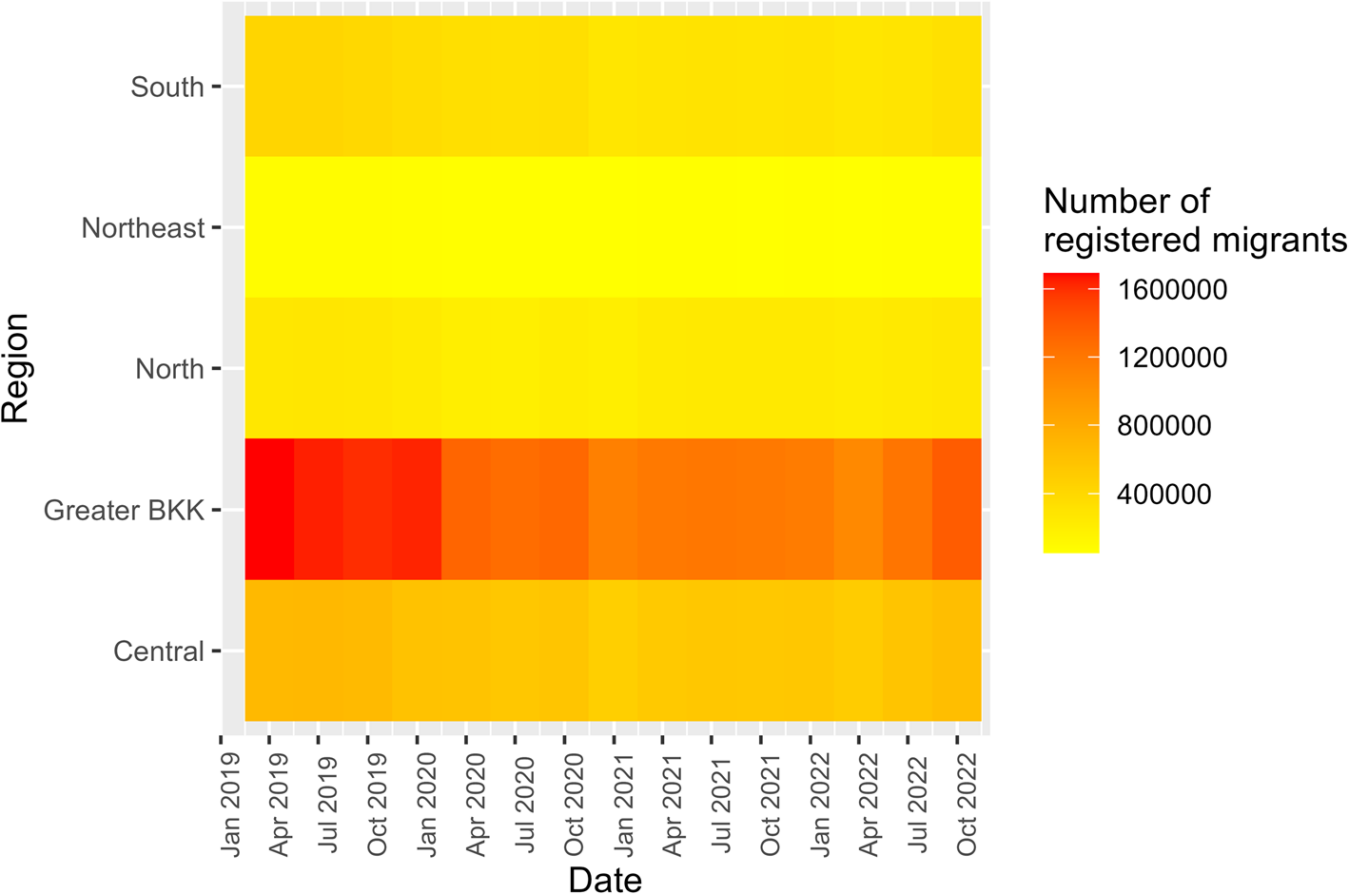
Prevalence number by registered migrants by regions, 2019–2022

Regarding regional distribution, Greater Bangkok consistently had the highest number of registered migrants, followed by the central, southern, northern, and northeastern regions. However, compared to 2019, all areas witnessed a decrease in the number of registered migrants after 2020. The decline ranged from 5.38% in the northern region and 9.84% in the central region to 18.93–20.67% in the other regions, Fig. 3.

**Fig. 4:**
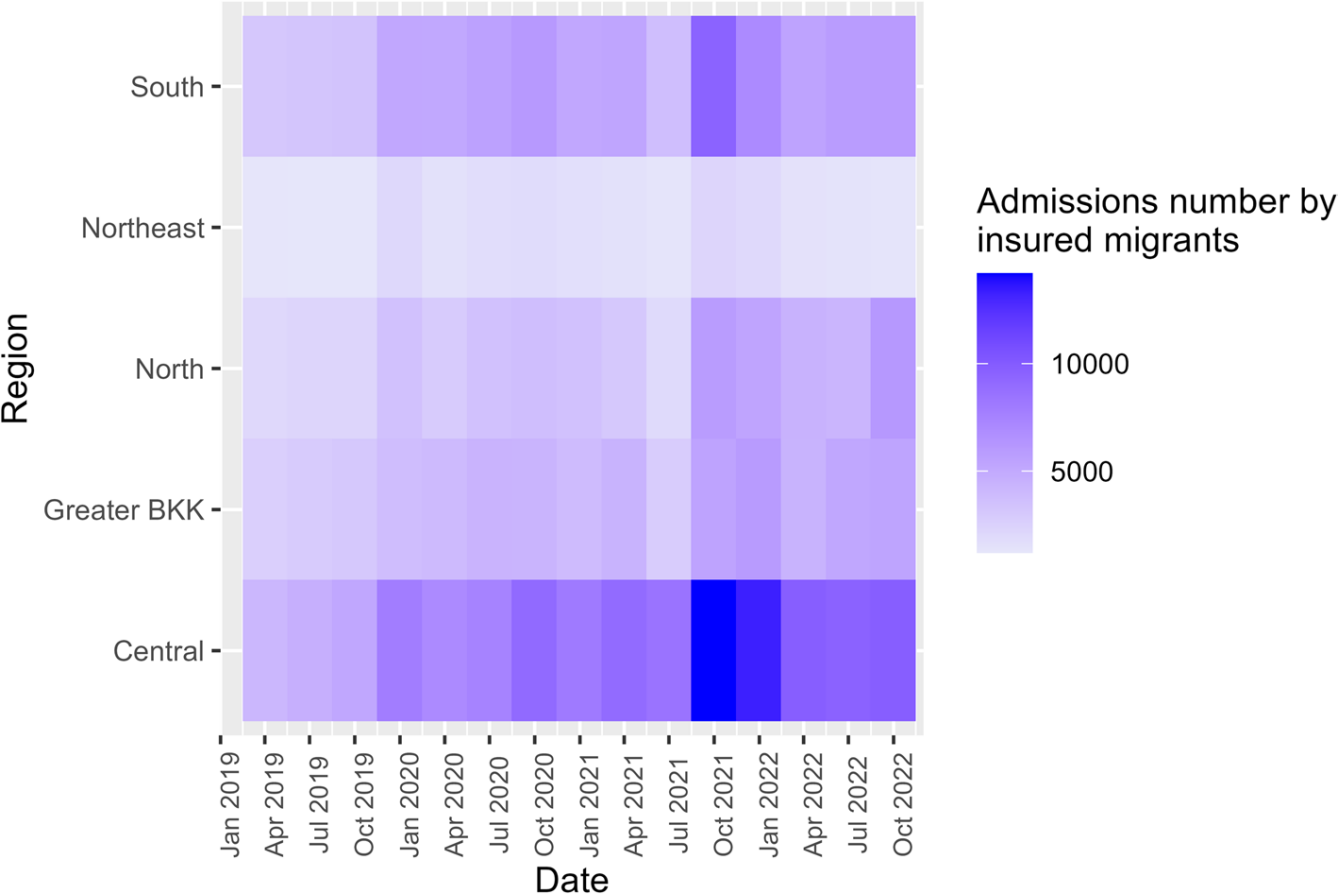
Admission number by insured migrants by regions, 2019–2022

For admission number amongst insured migrants, all regions saw an increase by 1.3–2.1 times after 2019, peaking in the final quarter of 2021 at the onset of the Omicron wave. The central region recorded the highest admission numbers (3,817–14,196 per quarter), while the northeastern region reported the lowest (1,205–2,251 per quarter), Fig. 4.

**Fig. 5:**
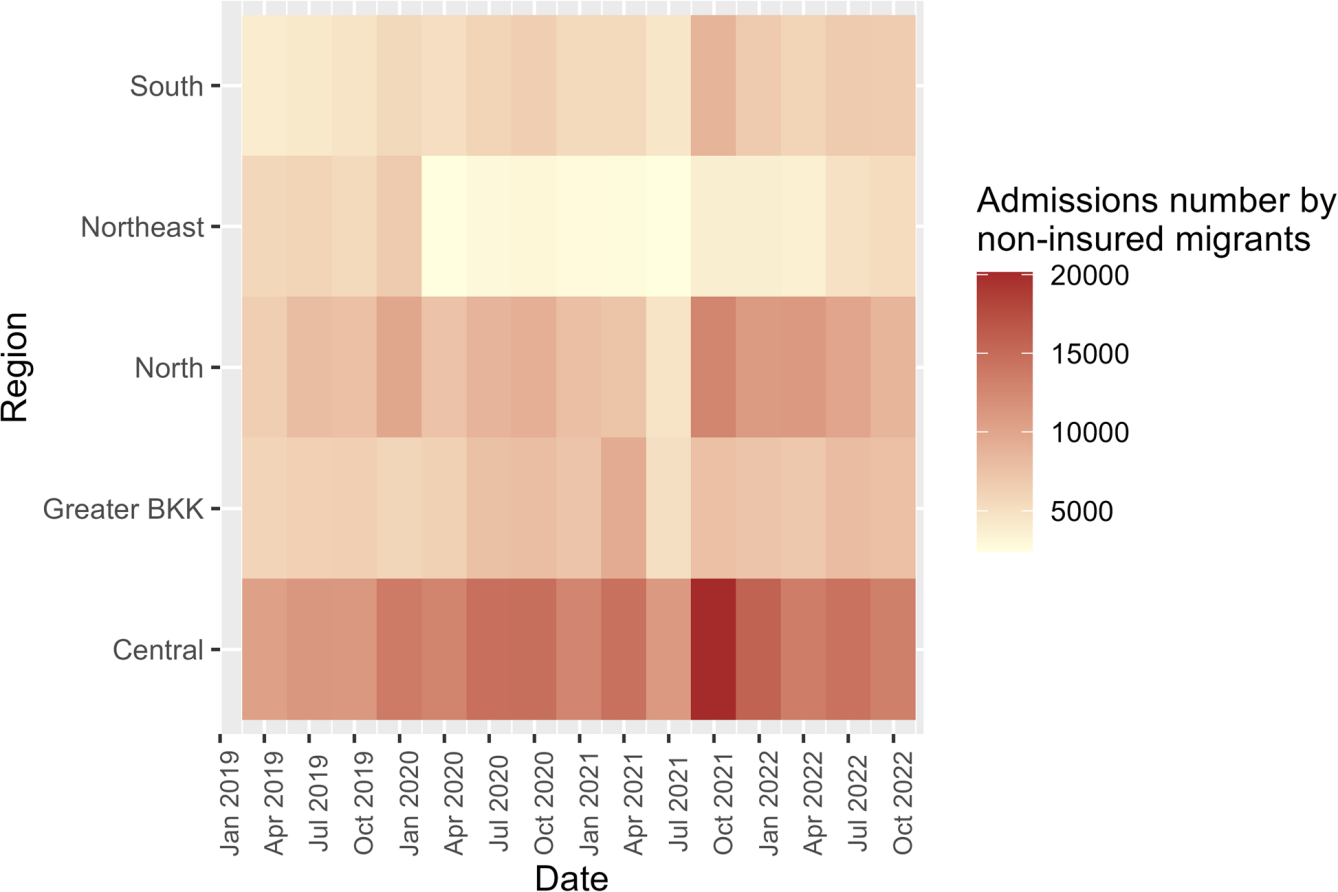
Admission number by non-insured migrants by regions, 2019–2022

The trend of admission numbers among non-insured migrants also mirrored that of insured migrants but with slightly higher figures. Once again, the central region had the highest numbers (10,530–20,139 per quarter), while the northeastern region saw the lowest (2,411–6,904 per quarter), Fig. 5.

**Fig. 6:**
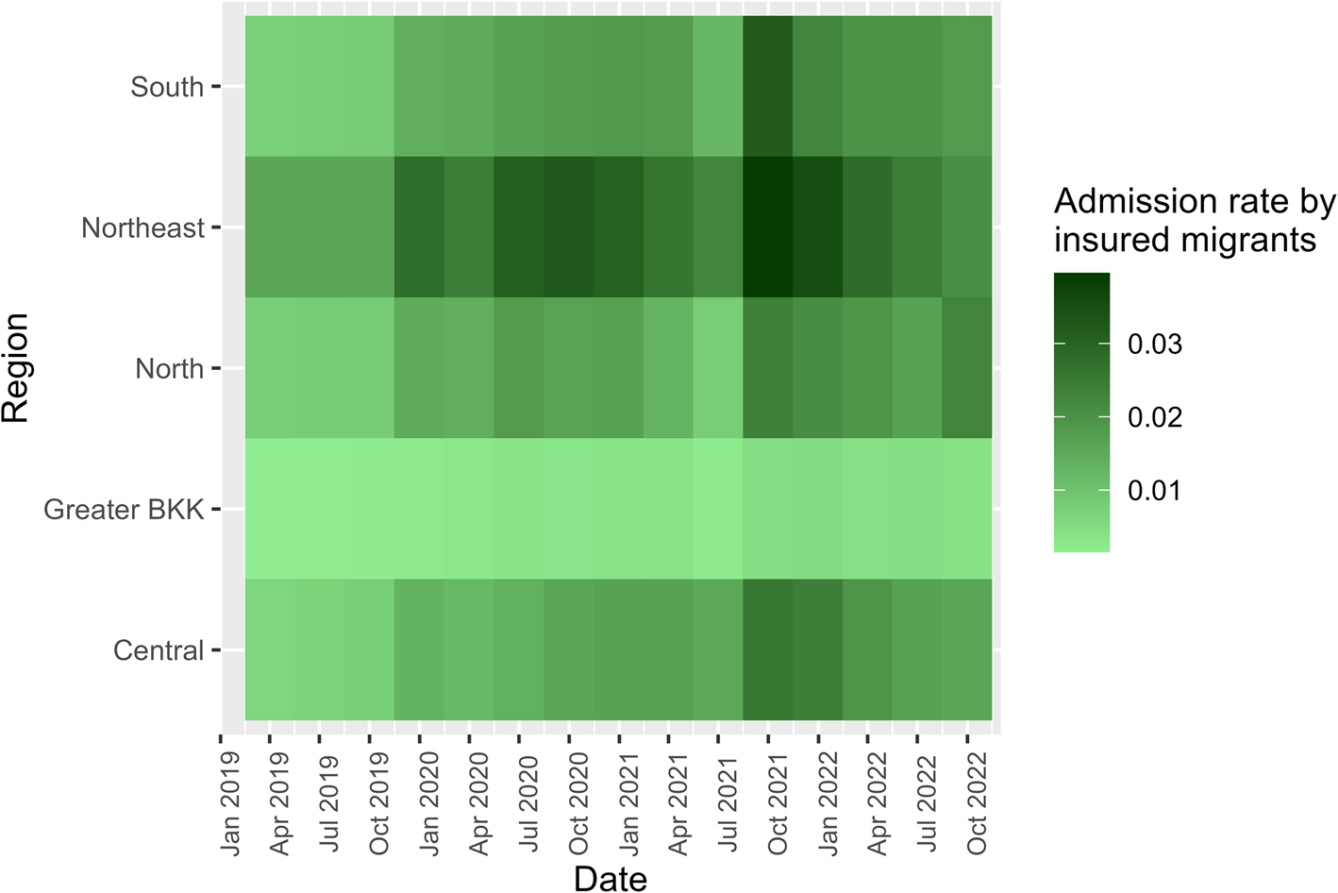
Admission rate by insured migrants by regions, 2019–2022

The admission rate displayed a similar temporal trend to the admission number, however, with the northeastern region having the highest rate (0.016–0.040 per quarter) and Greater Bangkok having the lowest rate (0.002–0.005 per quarter), Fig. 6.

Focusing on the COVID-19 period (2020–2022), the hospitalisation of migrants in public hospitals numbered between 137,826 and 184,630 individuals, resulting in 248,836 to 339,000 visits annually. The admissions by females outnumbered males in all years (male:female = 1:1.37 to 1:1.69). The mean age ranged from 23.79 to 26.36 years. Approximately 60% were non-insured migrants, while one-fifth utilised the SSS. Chiang Mai, Samut Sakhon, Kanchanaburi, Chon Buri, and Surat Thani consistently ranked among the top five provinces with the highest number of visits, Table 2.

**Table 2:** Number and characteristics of hospitalised migrants in public hospitals in Thailand, 2020–2022

Characteristics	2020	2021	2022
Total number of patients—n	137,826	184,630	159,001
Total number of visits—n	248,836	339,000	313,527
Male to female ratio	1:1.69	1:1.37	1:1.48
Mean age—years	23.79	26.35	25.54
Median age—years (P25-P75)	26 (1–35)	28 (20–36)	27 (7–36)
Percentage distribution of insurance status by visits—%			
• Health Insurance Card Scheme	8.55	7.72	9.02
• Social Security Scheme	15.78	22.65	17.53
• Others	12.23	12.95	14.94
• Non-insured	63.44	56.67	58.51
Top five provinces with largest visit number—%			
	Chiang Mai (8.36)	Samut Sakhon (10.50)	Chiang Mai (7.22)
	Samut Sakhon (7.45)	Chon Buri (6.94)	Samut Sakhon (6.84)
	Kanchanaburi (7.15)	Chiang Mai (6.81)	Kanchanaburi (6.69)
	Chon Buri (6.08)	Kanchanaburi (6.06)	Chon Buri (6.55)
	Surat Thani (5.39)	Surat Thani (5.18)	Surat Thani (5.68)

**Fig. 7:**
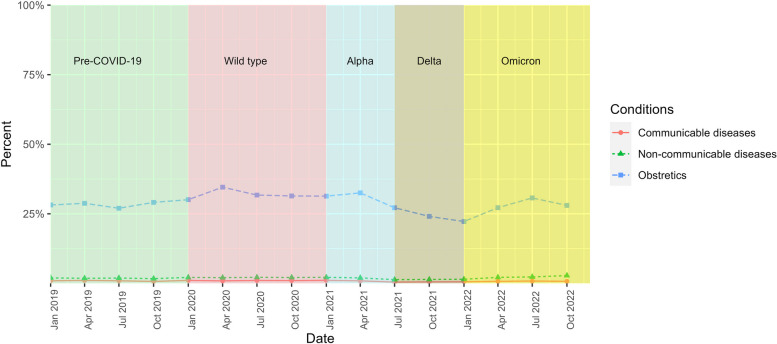
Percentage distribution of key disease groups amongst all admissions by insured migrants, 2019–2022

**Fig. 8:**
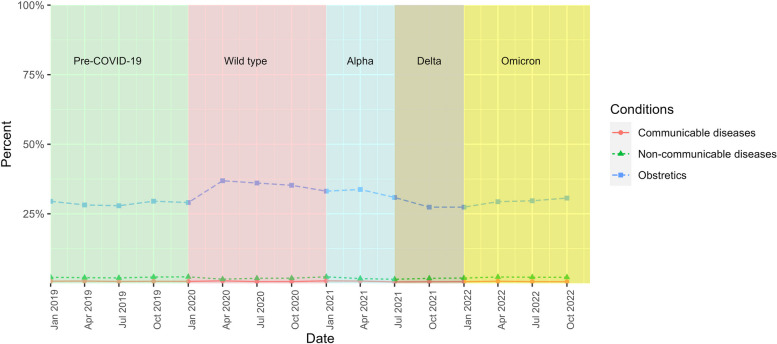
Percentage distribution of key disease groups amongst all admissions by non-insured migrants, 2019–2022

The admissions for selected communicable and non-communicable diseases and obstetric care remained consistent for both insured and non-insured migrants throughout all COVID-19 waves. Obstetric care exhibited the highest percentage share, with an approximate mean of 28.84% (22.23–34.58%) for insured migrants and 30.77% (27.39–36.88%) for non-insured migrants. Meanwhile, the mean share of communicable and non-communicable diseases stood approximately at 0.82% and 1.94% for insured migrants, and 0.71% and 1.98% for non-insured migrants, respectively, Figs. 7, and 8.

### Analytic study

Table 3 displays the outcomes of a random-effects negative binomial regression analysis examining the relationship between non-COVID-19 admissions, admission rates, and key variables, including the incidence of COVID-19 cases in the present and previous quarters, the number of hospital beds, regions of registration, and COVID-19 waves.

**Table 3:** Association between non-COVID-19 admissions and key predictor variables

Predictor variables	Outcome variables		
	Admission number by insured migrants—IRR^a^ (95% CI^b^)	Admission number by non-insured migrants—IRR^a^ (95% CI^b^)	Admission rate by insured migrants—IRR^a^ (95% CI^b^)
Increased incidence number of COVID-19 in the present quarter (1,000 persons)	1.00 (1.00, 1.00)	1.00 (1.00, 1.00)	1.00 (1.00, 1.01)^***^
Increased incidence number of COVID-19 in the previous quarter (1,000 persons)	1.00 (1.00, 1.00)	1.00 (1.00, 1.00)	1.00 (1.00, 1.01)^***^
The number of hospital beds (1,000 beds)	1.02 (0.99, 1.04)	0.95 (0.93–0.97)^***^	0.86 (0.83–0.89)^***^
Region (Greater Bangkok = ref)			
• North	0.92 (0.64, 1.32)	0.31 (0.22, 0.45)^***^	9.99 (7.28, 13.70)^***^
• Northeast	0.64 (0.45, 0.91)^*^	0.32 (0.22, 0.45)^***^	27.48 (19.69, 38.35)^***^
• Central	0.98 (0.68, 1.40)	0.60 (0.41, 0.87)^**^	5.68 (4.08, 7.91)^***^
• South	0.95 (0.65, 1.37)	0.64 (0.43, 0.94)^*^	6.69 (4.80, 9.32)^***^
Wave (Pre-COVID-19 = ref)			
• Wild type	1.51 (1.41, 1.62)^***^	1.19 (1.12, 1.27)^***^	1.78 (1.67, 1.89)^***^
• Alpha variant	1.44 (1.33, 1.57)^***^	1.15 (1.07, 1.24)^***^	1.94 (1.80, 2.09)^***^
• Delta variant	1.56 (1.43, 1.70)^***^	1.16 (1.07, 1.25)^***^	1.91 (1.76, 2.08)^***^
• Omicron	1.77 (1.65, 1.90)^***^	1.26 (1.19, 1.35)^***^	2.09 (1.96, 2.24)^***^

^*^P < *0.05*

^**^P < *0.01*

^***^P < *0.00*

^*a*^*Incidence rate ratio*

^b^*confidence interval*

For every 1,000 cases increase in the number of COVID-19 incidence, the admission numbers and rate remain unaffected, as displayed by the close-to-one IRR with a statistical significance observed in the rate analysis. As for each increment of 1,000 hospital beds, there was a statistically significant slight decrease in both the admission number for non-insured migrants and the admission rate for insured migrants. A negative relationship (IRR < 1) occurred between regional residence of registration and admission numbers. This relationship appeared to have statistical significance for insured migrants residing in the northeastern region and for non-insured migrants in all other regions. Conversely, Greater Bangkok saw the lowest admission rate amongst insured migrants, as reflected by large IRR values (varying between 5.68 and 27.48).

In the temporal angle, admission numbers and rates experienced significant increments across all COVID-19 waves compared with the pre-COVID-19 period. For insured migrants, the hospitalisation volume resulted in a percentage increase in numbers of 44.46–76.96%. For the non-insured migrants, the admission volume increased by 14.99- 26.35%. Notably, the admission rates approximately doubled in all waves, with the largest increase presented during the Omicron wave, Table 3. This analysis of migrant patients done separately for the three diagnostic groups (communicable, non-communicable diseases, and obstetric conditions) also shows a similar manner overall (Supplementary File 2).

## Discussion

To our knowledge, this study is amongst the few first studies, not only in Thailand but also in Asia, that investigated the trend of non-COVID-19 service before and during the pandemic and its associated factors. It is evident that the volume of non-COVID-19 admissions did not plummet during the surge of the COVID-19 pandemic. In contrast, it gradually soared as time passed, though some fluctuations existed around mid-2021. The sum of registered migrants and the volume of non-COVID-19 admissions was the largest in the central region. This is attributed to the region’s inclusion of industrial provinces on the eastern coast, hosting a vast number of migrants through a bilateral memorandum of understanding between Thailand and CLMV nations. Interestingly, Greater Bangkok showed the smallest value of admission rate despite having the highest prevalence number of insured migrants relative to other regions. This could be largely explained by the fall in the number of registered migrants in major cities from relocation to other regions and disruption in the work permit renewal process. During the pandemic, the prolonged outbreak and the contextual policy environment led to business shutdowns both temporarily and permanently. In November 2020, a rapid assessment on perceptions of non-Thai populations revealed that approximately 32% perceived widespread unemployment and financial difficulties, and 20% of respondents noted that more than three-quarters of non-Thai nationals in their communities had lost all sources of daily income due to COVID-19 [12]. This situation might influence the workers to relocate to other regions for job opportunities. Additionally, government services including the work permit renewal were hugely interrupted by numerous operational constraints surrounding the re-registration process [13, 14]. Such circumstances might cause some documented migrants to become undocumented. These undocumented migrants might scatter to other regions for informal working sectors instead of returning to their country of origin due to border crossing restrictions.

Concerning the findings in the analytic part, the change in the COVID-19 incidence cases (both the present value and the lag value) did not show any significant association with the non-COVID-19 admission volume amongst both insured and non-insured migrants. Focusing on the admission rate amongst insured migrants, though a statistical significance was observed in COVID-19 incidence, the association size was miniscule (about 0.5%). This phenomenon, on the one hand, mirrors the resilience of the healthcare system in the country that can absorb public health shock by maintaining IP care for patients in critical health need. Another evidence that indirectly points to health system resilience is the relative stability in the percentage share of key clinical conditions demonstrated in the figures above. This alludes to the fact that the share of services in various disease groups can be maintained regardless of the magnitude of the outbreak. A study by Wongtanasarasin et al. points in the same direction as our finding that the emergency department and intensive care unit admission rates increased by about 18% and 35%, respectively (comparing March to June 2020 with the same period in 2019) [15]. On the other hand, this finding should be interpreted with caution as it does not guarantee that the health need of the migrant population was met entirely. It is still possible that the enlargement in the admission number resulted from the disruption of routine OP services, and this might worsen the clinical conditions of non-severe patients—pushing them from OP care to IP care. This issue is supported by the gradual rise in admission volume in all COVID-19 waves (with a peak during the Delta wave) relative to the pre-COVID-19 period, while previous literature suggests a drop in the number of OP visits, though the degree of change varied by diseases and geographical areas. Sirikarn et al. observed a 26% fall in the monthly trend of the hospitalisation rate for epilepsy in the early COVID‐19 wave in Thailand. The most remarkable drop was found amongst children, whereas that amongst the older adults was trivial. Also, there was no significant change in hospitalisation rate in severe presentation of epilepsy like status epilepticus [16]. Sukmanee et al. reported a significant decline in the number of OP visits of the patients under the Universal Coverage Scheme—the main insurance arrangement for most Thai citizens—for many diagnostic groups during the lockdown period in 2020. The largest drop in OP utilisation was noticed in respiratory diseases (ICD-10: J00–J99). Meanwhile, the numbers of OP visits for metabolic diseases (ICD-10: E00–E90), diseases of the circulatory system (I00–I99), and obstetric conditions (ICD-10: O00-O99) during the post-lockdown period were not significantly different from the pre-pandemic period [17]. Arsenault et al. investigated the effect of COVID-19 on service disruption in ten countries (Chile, Ethiopia, Ghana, Haiti, Lao People’s Democratic Republic, Mexico, Nepal, South Africa, South Korea and Thailand). They found no clear disruption pattern by country income group or pandemic intensity [18]. In addition, various choices of treatment from the non-governmental healthcare sector might be one of the factors that impact hospital visits. A previous study on health-seeking preferences among immigrant workers in Songkhla province of Thailand showed that self-medication, private clinic, and factory clinic were majorly preferred when the workers had gastrointestinal and respiratory problems [19]. Myanmar migrants in Thailand from other studies were less likely to visit public hospitals or regulated healthcare facilities [20, 21]. Self-medication such as buying poly-pharmaceutical packs from grocery shop and taking left-over or herbal medicine at home, and private clinic were the usual choices. Additionally, some migrants also sought traditional healers and unqualified health workers [21]. To further explore this notion, a more in-depth analysis of individual-level data to track the change of clinical severity between pre-pandemic and amidst the pandemic for both OP and IP care is recommended. Future study exploring the utilization trend by illness category in more detailed, such as neurological, hematological diseases, and surgical conditions, would provide specific insights to and facilitate the tailored preparation of services for diverse patient groups.

Concerning geographical difference, for admission volume, almost all regions (except the northeastern region) shouldered nearly the same number of admissions compared with Greater Bangkok, as evidenced by an IRR being close to unity, even though the number of registered (insured) migrants in an individual province in Greater Bangkok is far greater than in other regions. A stark contrast in the size of the registered migrant population between Greater Bangkok and other regions with relatively similar hospitalisation volume across regions undoubtedly engenders the utilisation rate of IP care in Greater Bangkok far lower than in other areas. Sukmanee et al. discovered that the number of monthly OP visits in Bangkok was approximately four-fold lower than that of other health regions over four years (2017–2020) [17]. Another plausible explanation for the low admission rate in Greater Bangkok is the fragmentation of the primary care system. The primary care network in the upcountry is mainly organised by district health systems, comprising subdistrict health centres (health promoting hospitals), district hospitals, and provincial hospitals, whereas in Bangkok, there are no public district hospitals that have a clear mandate to take care of the residents in their catchment area. In Greater Bangkok, there exist a large number of private healthcare facilities and super-tertiary hospitals where their data recording system is disintegrated with the MOPH system [22]. It is possible that the IP utilisation rate amongst insured migrants in Greater Bangkok was indeed high as migrants might utilise services at private facilities, but their records were not shown in the MOPH data.

Methodology-wise, this study contains both strengths and weaknesses. The use of nationwide routine service data throughout the whole span of the pandemic can be viewed as one of the key strengths of this study. Another strong point is the application of random-effects negative binomial regression that helps account for spatio-temporal variation. However, certain limitations remain. First, as per the nature of the ecological study, ecological fallacy is inevitable. The analysis at the macro (provincial) level may not necessarily reflect the picture at the sub-provincial levels, let alone the individual patient level. Second, during the pandemic, each province was allowed to implement its own control measures as long as they were in line with the national policies as per the approval by the Provincial Governors. Details of the measures could be varied by province, and share of healthcare resources was common during the crisis. To this end, there might be unobserved confounders missing from the analysis. Besides, the cases emerging in one province might be hospitalised in nearby provinces. Hence, in our study, the observations in our dataset are not totally independent from one another. Albeit we attempted to address this issue by including region and time variables in the model, the interdependency between observations may still affect the validity of the analysis. Third, we lacked data on the service in private health facilities. This might undermine our ability to estimate the absolute magnitude of hospitalisation amongst migrants, especially in major cities where private hospitals hold a significant share of healthcare resources. Nonetheless, the main objective of this study is not to obtain the exact estimate of the service magnitude but to explore the changes in service volume and factors related to the changes; thus, this issue might not severely compromise our finding validity. The unlink of the service data between the MOPH data lake and private entities is not just a limitation for our study but also considers room for improvement for the health information system for the country as a whole. Fourth, the registration status and the insurance status of migrants are fluid. Albeit, in theory, all migrants registered for a work permit are obliged to be insured. In practice, the work permit valid date and the insurance expiry are not always well synced. Some non-insured migrants (with unknown numbers) showing up at health facilities might indeed obtain a work permit (being registered migrants). Vice versa, some work permit holders might let their insurance expire and become non-insured. Therefore, the admission rate in this matter should be cautiously interpreted, not to mention that a migrant registered to work in a province might utilise services in another province. We postulated that these measurement errors occur with random regardless of time and space (non-differential misclassification). Thus, the results in the final model are likely to be more conservative than the scenario where measurement errors did not exist. This issue also flags a dire need for inter-ministerial coherence to finetune and sync the MOL (work permit) data with the MOPH (service) data. Last but not least is the scarcity of research on migrant health relative to the Thai population. Most supporting literature demonstrated in the discussion above was based on the service behaviour of Thai citizens. An in-depth investigation on the health-seeking behaviour of migrant populations from both qualitative and quantitative angles is of huge value.

## Conclusion

The admission numbers and rates for non-COVID-19 healthcare services remained consistent regardless of the COVID-19 incidence numbers. As time passed by, the admission numbers and rates gradually increased. Greater Bangkok saw the lowest admission rates compared to all other regions. The increase in admission numbers and rates during the pandemic, on the one hand, might be caused by disruptions in OP service, leading to more severe cases seeking hospitalisation, but, on the other hand, mirrored the resilience of the healthcare system in Thailand in addressing public health crisis. The low admission rate amongst the insured migrants in Greater Bangkok could be partly attributed to the relatively low number of public service utilisation in stark contrast with the large number of registered migrants (despite a declining trend in the number of registered migrants over time) in relation to other regions. We recommend future studies that explore the healthcare-seeking behaviour of migrant populations at an individual level, using both quantitative and qualitative approaches. Such investigations will provide a better understanding of healthcare utilisation patterns amongst both registered and undocumented migrants, and ultimately lead to optimal policy design for migrant health as a whole.

### Supplementary Information

**Additional file 1:** **Table S1.** Characteristics of Social Security Scheme and Health Insurance Card Scheme for cross-border migrant workers in Thailand.

** Additional file 2:** **Table S2.** Association between admission number and admission rate of migrant patients with communicable, non-communicable diseases, and obstetric conditions and key predictor variables.

## Data Availability

The datasets used and/or analysed during the current study are available from the corresponding author upon reasonable request.

## Abbreviations

95% CI: 95% Confidence interval
CLMV: Cambodia, Lao PDR, Myanmar, and Vietnam
COVID-19: Coronavirus Disease 2019
ICD-10: International Classification of Diseases 10th Revision
IP: Inpatient
IRR: Incidence rate ratio
MOL: Ministry of Labour
MOPH: Ministry of Public Health
OP: Outpatient
SSS: Social Security Scheme
