# Supplementary material for: Utilisation of and factors associated with non-COVID-19 healthcare services in public facilities amongst cross-border migrants in Thailand, 2019–2022
Source: BMC Public Health. 2024 Jan 9;24:135. doi: 10.1186/s12889-024-17657-0 (PMC10777629; doi:10.1186/s12889-024-17657-0)
Supplement: Supplementary file 1 — Additional file 1: Table S1. Characteristics of Social Security Scheme and Health Insurance Card Scheme for cross-border migrant workers in Thailand. [file 12889_2024_17657_MOESM1_ESM.docx]

**Table S1** Characteristics of Social Security Scheme and Health Insurance Card Scheme for cross-border migrant workers in Thailand

| Characteristics | Health Care Scheme  (Governing body) | |
| --- | --- | --- |
|  | Social Security Scheme (Ministry of Labour) | Health Insurance Card Scheme  (Ministry of Public Health) |
| Eligibility criteria | Migrant workers in the formal sector | Migrant workers in the informal sector and dependants of migrant workers |
| Payment mechanism | Tri-partite payroll contribution by insuree, employer, and the Government (5% of monthly salary deducted) | Premium-based financing (1,600 Baht for a one-year coverage for migrants aged over 7 years plus 500 Baht medical screening before the enrolment and 365 Baht for a one-year coverage for migrants aged not more than 7 years) |
| Treatment benefit | Covering a wide range of care from outpatient service, inpatient service and high-cost care (disease prevention and health promotion benefit entrusted to the Universal Coverage Scheme—the main insurance arrangement for Thai citizens) | Covering a wide range of care from outpatient service, inpatient service, high-cost care, disease prevention and health promotion |
| Non-treatment benefit | Maternity leave allowance, unemployment compensation, disability compensation, death allowance and funeral expense compensation, childbirth allowance, and lump sum payment upon retirement (55 years of age) | None |
| Contracted facilities | Public hospitals and some contracted private hospitals | Only public hospitals affiliated with the Department of Medical Services and the Office of Permanent Secretary, the Ministry of Public Health, and certain public hospitals under the Bangkok Metropolitan Administration |
