# Supplementary material for: Utilisation of and factors associated with non-COVID-19 healthcare services in public facilities amongst cross-border migrants in Thailand, 2019–2022
Source: BMC Public Health. 2024 Jan 9;24:135. doi: 10.1186/s12889-024-17657-0 (PMC10777629; doi:10.1186/s12889-024-17657-0)
Supplement: Supplementary file 2 — Additional file 2: Table S2. Association between admission number and admission rate of migrant patients with communicable, non-communicable diseases, and obstetric conditions and key predictor variables. [file 12889_2024_17657_MOESM2_ESM.docx]

**Table S2** Association between admission number and admission rate of migrant patients with communicable, non-communicable diseases, and obstetric conditions and key predictor variables

| Predictor variables | Outcome variables | | | | | | | | |
| --- | --- | --- | --- | --- | --- | --- | --- | --- | --- |
|  | Admission number by insured migrants IRR^§^ (95% CI^±^) | | | Admission number by non-insured migrants IRR (95% CI) | | | Admission rate by insured migrants IRR (95% CI) | | |
|  | CD^1^ | NCD^2^ | OB^3^ | CD | NCD | OB | CD | NCD | OB |
| Increased incidence number of COVID-19 in the present quarter (1,000 persons) | 0.99  (0.99, 1.00)* | 1.00  (1.00, 1.00) | 1.00  (0.99, 1.00)*** | 1.00  (0.99, 1.00) | 1.00  (1.00, 1.00) | 1.00  (1.00, 1.00)* | 0.99  (0.99, 1.00)* | 1.00  (0.99, 1.00) | 1.00  (1.00, 1.00)* |
| Increased incidence number of COVID-19 in the previous quarter (1,000 persons) | 1.01  (1.01, 1.01)*** | 1.00  (1.00, 1.01) | 1.00  (1.00, 1.00)* | 1.01  (1.00, 1.01)* | 1.00  (1.00, 1.00) | 1.00  (1.00, 1.00)** | 1.01  (1.01, 1.01)*** | 1.01  (1.00, 1.01)*** | 1.00  (1.00, 1.01)*** |
| The number of hospital beds (1,000 beds) | 1.04  (0.93, 1.17) | 1.07  (0.95, 1.20) | 1.02  (0.99, 1.05) | 0.96  (0.92, 1.01) | 1.02  (0.95, 1.10) | 0.94  (0.91, 0.96)*** | 0.92  (0.89, 0.96)*** | 0.93  (0.90, 0.97)*** | 0.91  (0.88, 0.94)*** |
| Region (Greater Bangkok = ref) |  |  |  |  |  |  |  |  |  |
| - North | 0.44  (0.22, 0.88)* | 0.87  (0.51, 1.48) | 0.80  (0.53, 1.21) | 0.25  (1.14, 0.47)*** | 0.27  (0.15, 0.48)*** | 0.36  (0.24, 0.54)*** | 7.12  (4.04, 12.53)*** | 14.51  (8.81, 23.91)*** | 9.80  (6.89, 13.93)*** |
| - Northeast | 0.16  (0.08, 0.32)*** | 0.79  (0.46, 1.36) | 0.46  (0.30, 0.69)*** | 0.23  (0.13, 0.43)*** | 0.39  (0.22, 0.69)** | 0.49  (0.33, 0.73)*** | 8.31  (4.75, 14.56)*** | 32.59  (19.80, 53.63)*** | 17.59  (12.28, 25.20)*** |
| - Central | 0.83  (0.43, 1.60) | 1.27  (0.77, 2.09) | 0.95  (0.64, 1.42) | 0.55  (0.29, 1.05) | 0.69  (0.38, 1.25) | 0.74  (0.50, 1.11) | 4.35  (2.54, 7.44)*** | 7.44  (4.64, 11.94)*** | 5.22  (3.66, 7.45)*** |
| - South | 0.76  (0.38, 1.52) | 1.59  (0.91, 2.78) | 1.44  (0.93, 2.22) | 0.56  (0.29, 1.10) | 0.59  (0.32, 1.10) | 0.66  (0.43, 1.00)* | 5.07  (2.89, 8.88)*** | 8.22  (5.03, 13.43)*** | 7.23  (5.01, 10.42)*** |
| Wave (Pre-COVID-19 = ref) |  |  |  |  |  |  |  |  |  |
| - Wild type | 1.48  (1.24-1.76)*** | 1.60  (1.38, 1.86)*** | 1.76  (1.63, 1.90)*** | 1.10  (0.94, 1.29) | 1.08  (0.96, 1.22) | 1.38  (1.30, 1.46)*** | 1.72  (1.47, 2.02)*** | 1.95  (1.71, 2.23)*** | 1.99 ( 1.86, 2.12)*** |
| - Alpha variant | 1.49  (1.21, 1.84)*** | 1.53  (1.28, 1.83)*** | 1.76  (1.61, 1.93)*** | 1.37  (1.15, 1.64)** | 1.20  (1.04, 1.39)* | 1.27  (1.18, 1.36)*** | 1.83  (1.51, 2.24)*** | 2.17  (1.85, 2.56)*** | 2.28  (2.11, 2.46)*** |
| - Delta variant | 0.84  (0.65, 1.09) | 1.15  (0.94, 1.41) | 1.54  (1.39, 1.70)*** | 0.84  (0.68, 1.05) | 0.87  (0.74, 1.04) | 1.16  (1.08, 1.26)*** | 1.06  (0.83, 1.35) | 1.58  (1.31, 1.90)*** | 1.88  (1.73, 2.06)*** |
| - Omicron | 1.19  (0.98, 1.44) | 1.70  (1.45, 1.98)*** | 1.79  (1.65, 1.94)*** | 1.07  (0.90, 1.26) | 1.37  (1.21, 1.55)*** | 1.22  (1.15, 1.31)*** | 1.49  (1.24, 1.78)*** | 2.43  (2.11, 2.80)*** | 2.16  (2.02, 2.32)*** |

***Note:*** *^§^Incidence rate ratio; ^±^confidence interval; ^*^P<0.05; ^**^P<0.01; ^***^P<0.001, ^1^communicable diseases, ^2^non-communicable diseases, ^3^obstetric conditions*
